# Soil mite communities (Acari, Mesostigmata) in pure stands on post-agricultural lands: does season matter?

**DOI:** 10.1007/s10493-024-00968-7

**Published:** 2024-12-05

**Authors:** Jacek Malica, Cezary K. Urbanowski, Krzysztof Turczański, Grzegorz Rączka, Agnieszka Andrzejewska, Maciej Skorupski, Jacek Kamczyc

**Affiliations:** 1 Department of Game Management and Forest Protection, Faculty of Forestry and Wood Technology, Poznań University of Life Sciences, 71D Wojska Polskiego Str., 60-625 Poznan, Poland; 2 Department of Botany and Forest Habitats, Faculty of Forestry and Wood Technology, Poznań University of Life Sciences, Wojska Polskiego 71D, 60-625 Poznan, Poland; 3 Department of Forest Management Planning, Faculty of Forestry and Wood Technology, Poznań University of Life Sciences, 71C Wojska Polskiego Str, 60-625 Poznan, Poland; 4 Department of Agricultural Chemistry and Environmental Biogeochemistry, Faculty of Agronomy, Horticulture and Biotechnology, Poznań University of Life Sciences, Wojska Polskiego 71F, 60-625 Poznan, Poland

**Keywords:** Afforestation, Forest biodiversity, Post-agricultural land, Soil fauna, Mesostigmata

## Abstract

Post-agricultural land differs from typical forest land in physical, chemical and biological features. In addition, the environment of this land type is determined, among other things, by the introduced tree species. These differences may be revealed by the biodiversity and abundance of the soil fauna. We analysed the abundance, species richness and diversity of different instars of mesostigmatid mites inhabiting three different habitat types on post-agricultural land (shaped by pure *Pinus sylvestris* L., *Tilia cordata* Mill. and *Betula pendula* Roth stands). We collected 288 soil samples from eight plots in three stands. The collection was conducted in July and October in two consecutive vegetation seasons (2021 and 2022) for Mesostigmata mites community. Soil characteristics (determination of soil group and analysis of physical and chemical properties of soil and litter) were done in July 2021. In total, 399 individuals (266 females, 50 males and 83 juveniles) were classified into 38 taxa (33 species, five genera). Most individuals belonged to the Parasitidae, Laelapidae and Veigaiidae families. The most abundant species were *Hypoaspis aculeifer* (Canestini) (21.6% of all recorded mites), *Veigaia nemorensis* (C.L.Koch) (7.8%) and *Trachytes aegrota* (C.L.Koch) (7.0%). Abundance, species richness and diversity were shaped by collection month and Fe content in soil. The abundance was influenced by N litter content and was significantly lower in *P. sylvestris* stand in July (0.57 ± 0.23; mean ± SE) than in *P. sylvestris* (2.17 ± 0.54) and *T. cordata* (2.15 ± 0.48) stands in October. Moreover, abundance in *P. sylvestris* stand in October was higher than in *B. pendula* stand in July (0.78 ± 0.26). Similarly, species richness was significantly lower in *P. sylvestris* stand in July than in *P. sylvestris* and *T. cordata* stands in October (2.17 ± 0.54 and 2.15 ± 0.48, respectively). Higher Shannon’s diversity of mite communities was reported in *P. sylvestris* stand in October (0.40 ± 0.10) than in *P. sylvestris* and *B. pendula* stands in July (0.12 ± 0.06 and 0.14 ± 0.08, respectively). Large fluctuations of abundance, species richness and diversity of soil mite communities in *P. sylvestris* and *B. pendula* stands between collection months give the insights for creating mixed stands on post-agricultural land. It is worth noticing that the wet season creates the most favourable living environment for mesostigmatid mites in *P. sylvestris* litter.

## Introduction

The area of forests in Poland increased between 1945 and 2012 from 21 to 30% (i.e. from 6,470,000 ha to 9,164,000 ha). Such growth occurred primarily through the afforestation of post-agricultural lands (Kaliszewski 2016), accounting for 55% of afforestation—1,477,000 ha (Krawczyk 2015). Afforested post-agricultural land differs in physical and chemical characteristics from typical forest land. The most important differences include a higher pH and a lower C:N ratio. This results from long-term agricultural use—repeated ploughing and fertilization. Ploughing is responsible for the transformation of the soil profile and fertilization for the chemical changes (Gorzelak 1996). Additional differences are generated by the fact that these lands have been deprived for many years of the influence of tree root systems, which stabilize the soil structure and enrich it biologically (Thoms et al. 2010). The greater biodiversity of bacteria and lower diversity of fungi in soils of post-agricultural lands may be triggered by higher availability of nutrients (Delgado-Baquerizo et al. 2017). Planting of trees on post-agricultural land shapes the succession process of herbaceous plants and shrubs. This process is long-lasting and depends on the fragmentation of habitats and the vicinity of inhabited stands (Ciurzycki et al. 2021). The diversity of different plant functional groups may be positively correlated with soil functions such as microbial biomass carbon, basal respiration, and carbon sequestration. Thus, with the ongoing succession of these groups on post-agricultural land, the parameters may improve (Heydari et al. 2020). Both land use history and soil type are, therefore, a set of abiotic factors that shape the living conditions of trees and soil organisms. The importance of soil biota in soil remediation processes is based on their contribution to the decomposition of dead organic matter. Fungi, bacteria, nematodes (Nematoda), springtails (Collembola) and soil mites (Acari) should be considered as the main groups of such organisms. Good bioindicators of changes in the soil environment are free-living mesostigmatid mites (Mesostigmata). They occur in large numbers in the soil and forest litter, lead a relatively sedentary lifestyle (Kamczyc et al. 2019) and are sensitive to pollution and degradation of forest areas (Manu et al. 2019). They hunt primarily on springtails, nematodes, potworms (Enchytraeidae) or insect larvae (Insecta) (Koehler 1999; Wissuwa et al. 2012). The structure of mesostigmatid mite communities depends on the particular conditions of habitat type, such as vegetation, age of the trees or human impact and abiotic factors. Mesostigmatid mites highlight the degree of anthropogenization in ecosystems, but this effect is especially pronounced in forests (Călugăr, 2021). Mesostigmatid mite communities are also subject to seasonal changes in species composition and abundance concerning temperature and precipitation (Salmane 2000; Kamczyc et al. 2022). Environmental conditions and seasonal dynamics are also expressed through changes in the relationships of different mesostigmatid developmental instars, which include adult (males, females) and three juvenile instars (deutonymphs, protonymphs and larvae) (Urbanowski et al. 2021).

The aim of the study was to recognize differences in seasonal changes in the abundance, species richness and diversity of Mesostigmata mite assemblages inhabiting pure forests (*Betula pendula* Roth*, Tilia cordata* Mill., *Pinus sylvestris* L.) growing on post-agricultural lands. Considering previous studies on post-agricultural lands (Scheu and Schulz 1996; Gormsen et al. 2006; Gawęda et al. 2021), we hypothesized that abundance, species richness, diversity, and relative proportion of different instars (including sex ratio) in mite communities would respond in different ways to (1) season (summer and autumn), (2) tree species, and (3) soil properties. We expected that various forest litter types would affect soil mite assemblages differently, and that effect would change with season.

## Materials and methods

### Site description and experimental design

The study site was located in the Opole Forest District (S–W Poland), where coniferous forests cover 60% of the forest area. The forests are dominated by mesic (57% of area), moist (40%), and marsh sites (~ 3%). Considering the soil environment, the main groups of soils are Arenosols (rusty soils)—45.3%, Podzols (podzolic soils)—23.0%, and Gleysols (gleyic soils)—16.4% (Forest Management Plan for Opole Forest Division, 2014). The vegetation season lasts 227 days. Mean annual precipitation reaches 603 mm, while mean annual temperature is 8 °C. The duration of winter is 60–70 days, and the number of days with snow cover is 58–65 days. In turn, the duration of summer is 90–100 days (Malica et al. 2024).

**Fig. 1:**
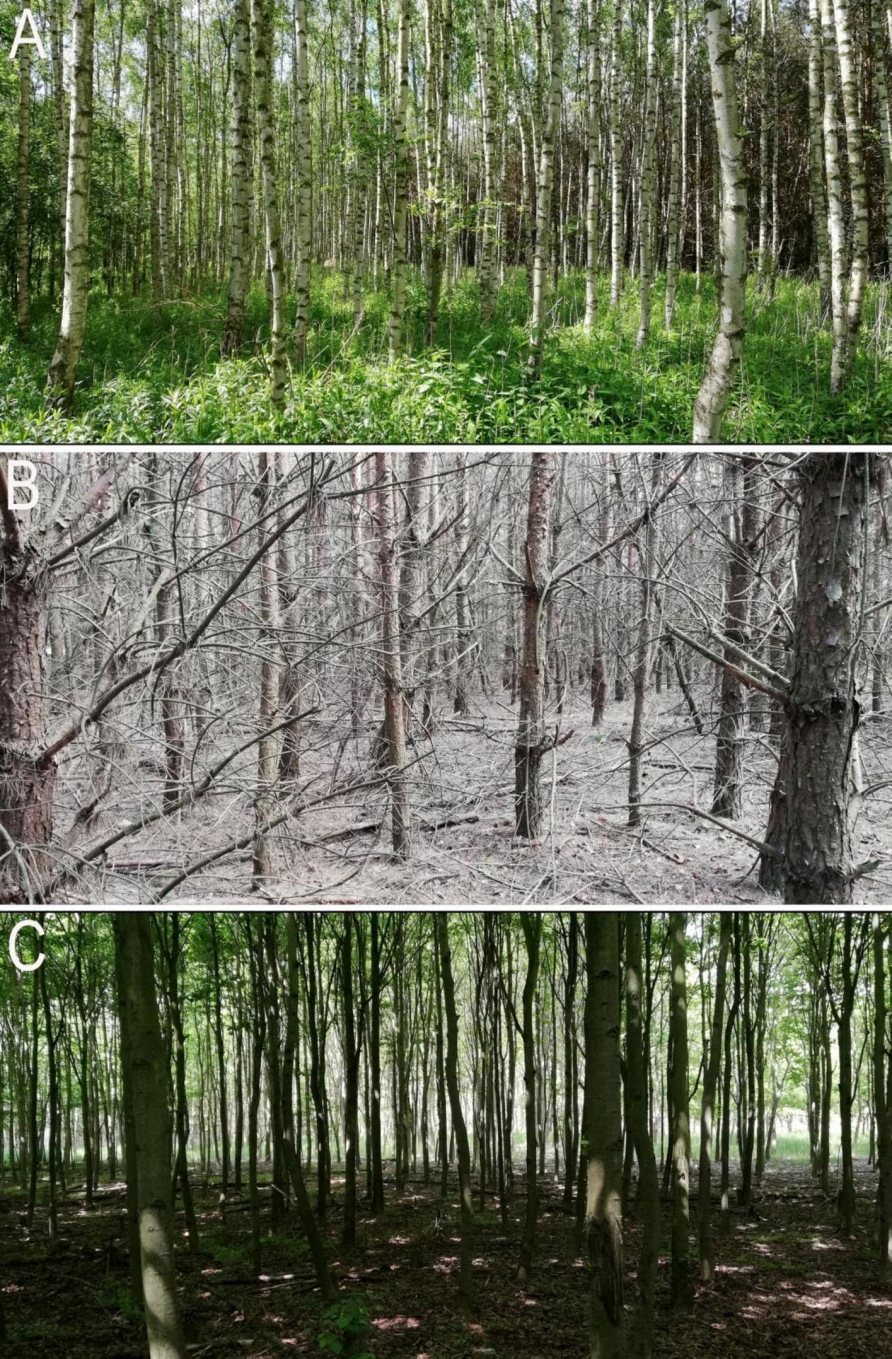
Study sites on post-agricultural lands (July 2021) in the Opole Forest District: **A** *Betula pendula* stand, **B** *Pinus sylvestris* stand, **C** *Tilia cordata* stand

At the beginning of our study, three pure stands of different tree species (*Pinus sylvestris* L., *Tilia cordata* Mill. and *Betula pendula* Roth) were selected (Fig. 1). Every stand was located on post-agricultural land characterized by sandy, acidic or slightly acidic soils, with no calcium carbonates and low soil organic matter content in upper soil horizons (rusty soil—Arenosol). The distance between stands was at least 50 m. Finally, eight circular study plots (0.02 ha) were established in total. The study site characteristics were done in summer 2021 to note all plant species growing on each site. On each study plot, we measured: (1) the total number of trees (N); (2) the height of the trees (m); (3) the dimension at breast height—DBH (cm). Moreover, all vascular plants were recorded and the cover of each species in each layer was estimated using the seven-level Braun-Blanquet abundance scale (Table 4 in Appendix 1).

### Soil analyses

The soil environment was characterized by the description of soil pit (at least to a depth of 1 m) located in the middle of the chosen stands. Subsequently, we described the reference soil groups based on the IUSS WRB (2015) and took soil and litter (mineral and organic layer separately) samples (ca. 500 g in total) from every soil horizon for further laboratory studies (16 samples in total). Soil samples were collected to determine: (1) soil texture (Casagrande’s aerometric method modified by Prószyński); (2) pH of soil and litter—measured in distilled water (potentiometric method); (3) content of organic carbon (Corg%—Tiurin’s method); (4) soil organic matter (SOM%—loss on ignition method); (5) the total nitrogen content (N%—Kjeldahl’s method); (6) C to N ratio; (7) elements such as Mg, Na, Ca, P, K (%) as well as Fe, Cd, Pb, Mn, Zn, and Cu (mg/kg) using atomic absorption spectrometry analysis using the AAS Varian 55B spectrometer (Peña et al. 2016). Moreover, we collected samples of intact soil for analysis of bulk density (BD; g/cm^3^)—core method, and actual soil moisture (SM; %)—gravimetric method (Lityński et al. 1976). The analyses were conducted in the laboratory at the Poznań University of Life Sciences (Table 5 in Appendix 2).

### Mesostigmata mites investigation

Sampling was carried out four times: in July and October 2021 and in July and October 2022. Collecting of the soil samples (288 samples in total = eight plots × nine samples per plot × four samplings) was conducted on each study plot using metal soil corer (ø 5 cm) to the depth of 15 cm. In the laboratory, samples were placed on the Berlese-Tullgren apparatus, consisting of a funnel, a light bulb (40W), a strainer (mesh size 2 mm), and containers with 75% ethanol solution. Edaphon was extracted from the soil samples for at least seven days (until the collected samples were dry). Mesostigmatid mite individuals were selected from the alcohol solution under a stereomicroscope and placed in the Hoyer’s medium on slides. The detailed diagnosis took place under a compound microscope Zeiss Axio Scope.A1. All individuals were classified into the appropriate instar, as well as species level or higher taxonomic unit using identification keys (Micherdziński 1969; Karg 1971, 1993; Gwiazdowicz 2007; Mašán & Halliday 2014).

### Data analysis

Sampled material allowed to determine mite abundance (ind.), species richness and Shannon–Wiener diversity index (H′ = − Σpiln(pi), where pi is the proportion of particular species in the mite community) for each of the plots. During analysis raw, data for abundance, species richness and diversity of mesostigmatid mite communities were presented as mean (± SE) values per sample within collection month and habitat type. In addition, for each species, indicators of dominance and constancy (the ratio of samples in which a given species occurs to all samples collected in a given variant) were determined for each habitat variant. Statistical analyses were done using R software (R version 4.1.2; R Core Team R: A Language and Environment for Statistical Computing; available online: https://www.r-project.org/). We used generalized linear models (GLM) assuming a Tweedie distribution for mesostigmatid abundance, species richness, and diversity calculated per sample and per plot. We applied predictors in models with low variance inflation factors (VIF < 5). Furthermore, we conducted Tukey post-hoc tests for each model to determine the differences in studied factors between month/habitat combinations. The statistical significance of variables used in GLM’s was calculated using t-values. Results were treated as significant when *p* < 0.05. In order to describe the relationship between mesostigmatid mite assemblages and habitat characteristics, including soil properties, we conducted canonical correspondence analysis (CCA) implemented in the *vegan* package in R. The variables included in the CCA final model were based on Akaike information criterion (AIC). Furthermore, environmental variables were selected on VIF < 2. To test the factor's significance, we conducted a permutational analysis of variance (PERMANOVA). We plotted a cumulative species number using the vegan::specaccum() function (Oksanen et al. 2018). Furthermore, we used the bipartite package to reveal the relationship between mesostigmatid mite taxa and habitats (Dormann et al. 2008). In the conducted bipartite interaction network we assumed habitats as lower-level and mite taxa as higher-level groups. The taxa level response to specific habitat was described by specialisation index d′, derived from the Kulback-Leibler distance, expressing whether a given taxon is a non-specialist or a perfect specialist (range from 0 to 1). Additionally, d′ index describes how strongly the taxa differ from a random sampling of available interacting partners (Dormann 2011).

## Results

### Overall results

**Fig. 2:**
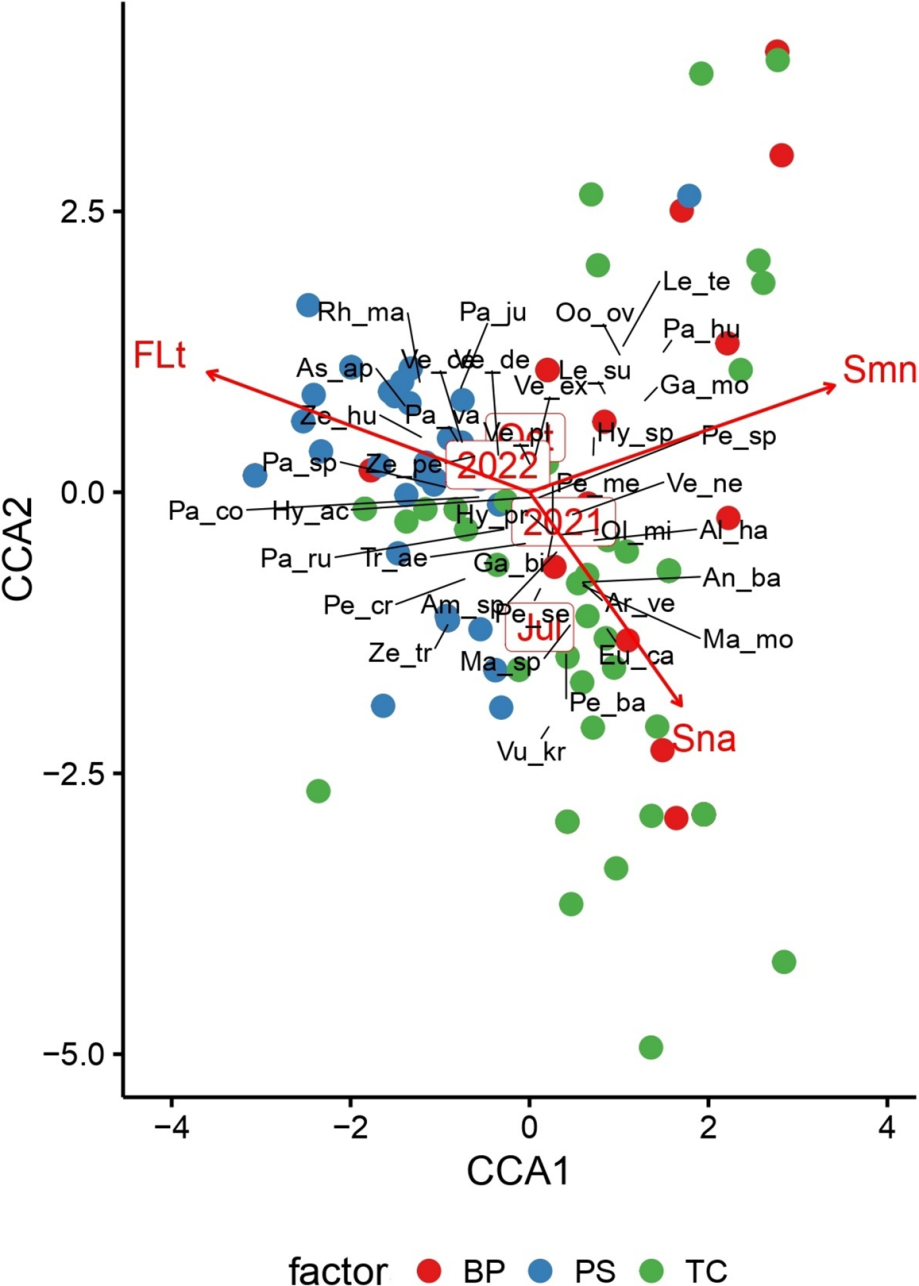
Canonical correspondence analysis (CCA) for Mesostigmata communities across four sampling periods (July, October; 2020, 2021) in pure stands on post-agricultural lands (Opole Forest District). Mite taxa labelled with the first two letters of the genus name and the first two letters of the species name for each mite taxon (for abbreviations of mite taxa see Table 6 in Appendix 3). *BP*—*B. pendula*, *PS*—*P. sylvestris*, *TC*—*T. cordata*, *Jul*—July, *Oct*—October, *FLt*—forest litter thickness, *Sna*—soil Na content, Smn—soil Mn content

In total, 399 individuals (266 females, 50 males and 83 juveniles) were classified as 38 taxa (33 species, five genera) (Table 1). The GLM models assuming Tweedie distribution revealed that collection month and Fe content in soil affected abundance, species richness and diversity. Moreover, mesostigmatid mite abundance was influenced by litter N content (Table 2). The canonical correspondence analysis (CCA) revealed significant differences in mite taxa distribution. Axis 1 and axis 2 explained 26.1% and 25.0% of variance in community composition, respectively (Fig. 2). Axis 1 reflects collection time, while Axis 2 reflects the tree species studied. The analysis of variance conducted for the model showed that collection month (*p* = 0.002), year (*p* = 0.002), soil Mn content (*p* = 0.001), soil Na content (*p* = 0.028) and litter thickness (*p* = 0.036) significantly influenced the mite communities structure (Table 3). Species of the genus *Paragamasus* were influenced by soil Mn content and litter thickness. In contrast, species belonging to the genus *Veigaia* were affected by soil Na content and were also the least sensitive to soil Mn among all other genera.

**Table 1:** Species composition, dominance, and constancy of occurrence of individual mite species in each type of tree stand

No.	Mite taxa	*Betula pendula*			*Tilia cordata*			*Pinus sylvestris*		
		Individuals number	Dominance (%)	Constancy	Individuals number	Dominance (%)	Constancy	Individuals number	Dominance (%)	Constancy
1	*Alliphis halleri* (Canestrini & Canestrini, 1881)	0	0.00	0.00	1	0.58	0.01	0	0.00	0.00
2	*Amblyseius* spp.	1	1.27	0.01	5	2.91	0.05	0	0.00	0.00
3	*Antennoseius bacatus* Athias-Henriot, 1961	1	1.27	0.01	1	0.58	0.01	0	0.00	0.00
4	*Arctoseius venustulus* (Berlese, 1917)	1	1.27	0.01	1	0.58	0.01	0	0.00	0.00
5	*Asca aphidioides* (Linnaeus, 1758)	0	0.00	0.00	0	0.00	0.00	4	2.70	0.05
6	*Eugamasus cavernicola* (Trägårdh, 1912)	0	0.00	0.00	4	2.33	0.04	0	0.00	0.00
7	*Gamasellodes bicolor* (Berlese, 1918)	0	0.00	0.00	1	0.58	0.01	0	0.00	0.00
8	*Gamasellus montanus* (Willman, 1936)	1	1.27	0.01	1	0.58	0.01	0	0.00	0.00
9	*Hypoaspis* (*Gaeolaelaps*) *aculeifer* (Canestrini, 1883)	16	20.25	0.17	41	23.84	0.31	29	19.59	0.22
10	*Hypoaspis* spp.	2	2.53	0.01	5	2.91	0.02	0	0.00	0.00
11	*Hyposapis* (*Gaeolaelaps*) *praesternalis* (Willmann, 1949)	0	0.00	0.00	2	1.16	0.01	0	0.00	0.00
12	*Leptogamasus suecicus* (Trägårdh, 1936)	5	6.33	0.03	0	0.00	0.00	0	0.00	0.00
13	*Leptogamasus tectegynellus* (Athias Henriot, 1967)	0	0.00	0.00	2	1.16	0.01	0	0.00	0.00
14	*Macrocheles montanus* (Willmann, 1951)	4	5.06	0.04	8	4.65	0.06	0	0.00	0.00
15	*Macrocheles* spp.	2	2.53	0.01	0	0.00	0.00	0	0.00	0.00
16	*Olodiscus minima* (Kramer, 1882)	3	3.80	0.04	3	1.74	0.04	2	1.35	0.02
17	*Oodinychus ovalis* (C.L.Koch, 1839)	10	12.66	0.03	11	6.40	0.05	1	0.68	0.01
18	*Pachyseius humeralis* (Berlese, 1910)	0	0.00	0.00	1	0.58	0.01	0	0.00	0.00
19	*Paragamasus conus* (Karg 1971))	1	1.27	0.01	12	6.98	0.11	13	8.78	0.11
20	*Paragamasus jugincola* (Athias Henriot, 1967)	0	0.00	0.00	0	0.00	0.00	1	0.68	0.01
21	*Paragamasus runcatellus* (Berlese, 1903 sensu Karg 1971)	5	6.33	0.04	2	1.16	0.02	7	4.73	0.07
22	*Paragamasus* spp.	1	1.27	0.01	3	1.74	0.01	12	8.11	0.10
23	*Paragamasus vagabundus* (Karg, 1968)	2	2.53	0.03	1	0.58	0.01	19	12.84	0.11
24	*Pergamasus barbarus* (Berlese, 1904)	0	0.00	0.00	4	2.33	0.04	1	0.68	0.01
25	*Pergamasus crassipes* (Linnaeus, 1758)	0	0.00	0.00	1	0.58	0.01	1	0.68	0.01
26	*Pergamasus mediocris* Berlese, 1904	0	0.00	0.00	2	1.16	0.00	0	0.00	0.00
27	*Pergamasus septentrionalis* (Oudemans, 1902)	1	1.27	0.01	6	3.49	0.04	2	1.35	0.01
28	*Pergamasus* spp.	1	1.27	0.01	2	1.16	0.02	1	0.68	0.01
29	*Rhodacarus mandibularis* (Berlese, 1921)	0	0.00	0.00	0	0.00	0.00	1	0.68	0.01
30	*Trachytes aegrota* (C.L.Koch, 1841)	0	0.00	0.00	18	10.47	0.11	10	6.76	0.10
31	*Veigaia cerva* (Kramer, 1876)	0	0.00	0.00	0	0.00	0.00	1	0.68	0.01
32	*Veigaia decurtata* (Athias Henriot, 1961)	1	1.27	0.01	2	1.16	0.01	7	4.73	0.04
33	*Veigaia exigua* (Berlese, 1916)	11	13.92	0.07	5	2.91	0.05	10	6.76	0.09
34	*Veigaia nemorensis* (C.L.Koch, 1839)	9	11.39	0.08	18	10.47	0.19	4	2.70	0.05
35	*Veigaia planicola* (Berlese, 1892)	1	1.27	0.01	0	0.00	0.00	1	0.68	0.01
36	*Vulgarogamasus kraepelini* (Berlese, 1904)	0	0.00	0.00	1	0.58	0.01	0	0.00	0.00
37	*Zercon peltatus* (C.L.Koch, 1836)	0	0.00	0.00	8	4.65	0.06	18	12.16	0.16
38	*Zercon hungaricus* (Sellnick, 1958)	0	0.00	0.00	0	0.00	0.00	2	1.35	0.01
39	*Zercon triangularis* (C.L.Koch, 1836)	0	0.00	0.00	‍0	0.00	0.00	1	0.68	0.01

**Table 2:** Generalized linear models assuming a Tweedie distribution explaining mite abundance, species richness, and Shannon’s diversity

Abundance									Species richness									Diversity						
Term	Estimate		SE		t value		Pr( >|t|)		Estimate		SE		t value		Pr( >|t|)			Estimate		SE		t value		Pr( >|t|)
(Intercept)	24.8773		11.9204		2.087		**0.049**		24.4813		9.8568		2.484		**0.021**			27.1233		12.6038		2.152		**0.043**
tree_PS	− 0.9236		1.1251		− 0.821		0.421		− 0.4704		0.9547		− 0.493		0.627			− 0.3591		1.2443		− 0.289		0.776
tree_TC	− 0.2537		0.5448		− 0.466		0.646		− 0.1508		0.4757		− 0.317		0.754			− 0.0808		0.6561		− 0.123		0.903
coll_month	0.8957		0.2284		3.922		** < 0.001**		0.6762		0.1878		3.600		**0.002**			0.7645		0.2437		3.137		**0.005**
year_2022	0.0346		0.2145		0.161		0.874		− 0.0606		0.1777		− 0.341		0.736			− 0.1496		0.2275		− 0.658		0.518
som	− 0.5677		0.4301		− 1.320		0.201		− 0.5964		0.3610		− 1.652		0.113			− 0.6977		0.4660		− 1.497		0.149
fl_n	2.0189		1.2076		1.672		0.109		2.1843		1.0003		2.184		**0.040**			2.6937		1.2910		2.086		**0.049**
ph_litter	− 0.7052		0.9492		− 0.743		0.465		− 0.4079		0.7937		− 0.514		0.613			− 0.5508		1.0080		− 0.546		0.590
fl_ca	− 2.7421		2.8281		− 0.970		0.343		− 3.5976		2.4797		− 1.451		0.161			− 4.3148		3.4930		− 1.235		0.230
soil_fe	− 0.0665		0.0319		− 2.087		**0.049**		− 0.0714		0.0259		− 2.752		**0.012**			− 0.0829		0.0324		− 2.556		**0.018**

Analysis of Deviance (Type III tests)									
Term	LR Chisq	Df	Pr(> Chisq)	LR Chisq	Df	Pr(> Chisq)	LR Chisq	Df	Pr(> Chisq)
tree	0.7028	2	0.704	0.2566	2	0.880	0.0855	2	0.958
coll_month	16.1624	1	** < 0.001**	13.7086	1	** < 0.001**	10.5735	1	**0.001**
year	0.0258	1	0.872	0.1165	1	0.733	0.4336	1	0.510
som	1.7972	1	0.180	2.8857	1	0.089	2.3942	1	0.122
fl_n	2.8380	1	0.092	4.9247	1	**0.027**	4.5450	1	**0.033**
ph_litter	0.5553	1	0.456	0.2657	1	0.606	0.3021	1	0.583
fl_ca	0.9459	1	0.331	2.1511	1	0.143	1.5817	1	0.209
soil_fe	4.3780	1	**0.036**	7.7242	1	**0.006**	6.8199	1	**0.009**

*SE*—standard error, *tree_PS*—*Pinus sylvestris*, *tree_TC*—*Tilia cordata*, *coll_month*—collection month, *year*—year of sampling, *som*—soil organic matter, *fl_n*—litter N content, *ph_litter*—litter pH, *fl_ca*—litter Ca content, *soil_fe*—soil Fe content

**Table 3:** The influence of environmental variables on Mesostigmata communities (PERMANOVA)

Term	Df	ChiSquare	F	Pr(> F)
soil_mn	1	0.2707	1.9727	**0.001**
coll_month	1	0.2490	1.8147	**0.002**
year	1	0.2306	1.6803	**0.002**
fl_thickness	1	0.1991	1.4506	**0.036**
soil_na	1	0.2066	1.5054	**0.028**
Residual	125	17.1526		
Model	5	1.1559	1.6847	**0.001**
Residual	125	17.1526		

*soil_mn* soil Mn content, *coll_month* collection month, *year* year of collection, *fl_thickness* forest litter thickness, *soil_na* soil Na content

### Seasonal changes in mesostigmatid mite communities

**Fig. 3:**
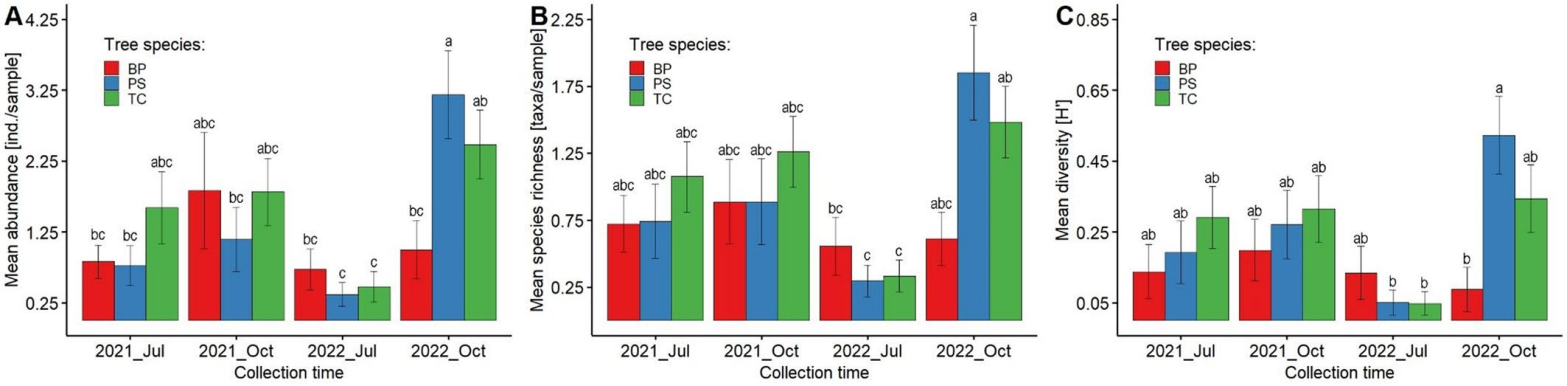
Mean abundance (**A**), species richness (**B**) and Shannon’s diversity (**C**) of mesostigmatid mites in the total communities across four sampling periods (July, October; 2020, 2021) in pure stands on post-agricultural lands (Opole Forest District). Abbreviations of variants: *Oct*—October, *Jul*—July, *BP*—*B. pendula*, *PS*—*P. sylvestris*, *TC*—*T. cordata*, *Oct_PS*—October/*P. sylvestris*, *Jul_PS*—July/*P. sylvestris*, *Oct_TC*—October/*T. cordata*, *Jul_BP*—July/*B. pendula*, *Jul_TC*—July/*T. cordata*, *Oct_BP*—October/*B. pendula*

The highest abundance calculated per sample was recorded in *P. sylvestris* stand in October 2022 (3.19 ± 0.62 ind.), while the lowest was recorded in *P. sylvestris* stand in July 2022 (0.37 ± 0.17). The highest abundance in July was reported in *T. cordata* stand in 2021 (1.59 ± 0.50 ind.) (Fig. 3A). The Tukey post-hoc tests revealed that mesostigmatid mite abundance was significantly lower in *P. sylvestris* stand in July (0.57 ± 0.17 ind.) than in *P. sylvestris* and *T. cordata* stands in October (2.17 ± 0.41 and 2.15 ± 0.34, respectively). Moreover, abundance in *P. sylvestris* stand in October was significantly higher than in *B. pendula* stand in July (0.78 ± 0.18 ind.).

The highest species richness per sample was recorded in *P. sylvestris* stand in October 2022 (1.85 ± 0.35 species) and the lowest in *P. sylvestris* stand in July 2022 (0.30 ± 0.12) (Fig. 2B). Species richness of mesostigmatid mite communities was significantly lower in *P. sylvestris* stand in July (0.52 ± 0.15 species) than in *P. sylvestris* and *T. cordata* stands in October (1.37 ± 0.25 and 1.37 ± 0.19, respectively) (Fig. 3B).

The highest mesostigmatid mite Shannon’s diversity was found in *P. sylvestris* stand in October 2022 (0.52 ± 0.11), while the lowest was in *T. cordata* stand in July 2022 (0.05 ± 0.03). A significantly higher diversity was found in *P. sylvestris* stand in October (0.40 ± 0.07) compared to *P. sylvestris* and *B. pendula* stands in July (0.12 ± 0.05 and 0.14 ± 0.05, respectively) (Fig. 3C).

### Mite communities structure

**Fig. 4:**
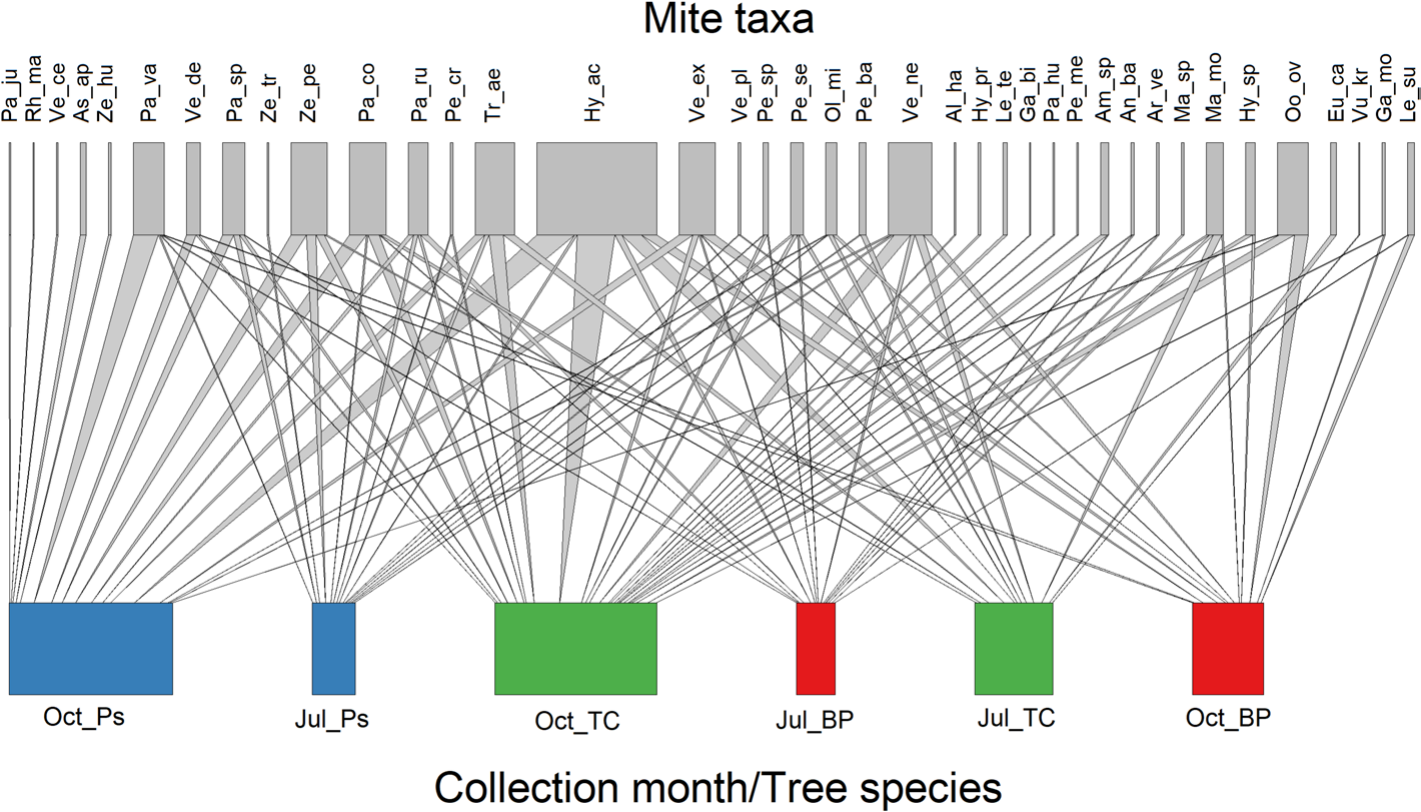
Co-occurrence network for bipartite relationships between mesostigmatid mite taxa (upper boxes) and collection Month/Habitat (lower boxes). Boxes are proportional to total mite abundance, whereas ribbon width is proportional to the co-occurrence. *Oct_PS*—October/*P. sylvestris*, *Jul_PS*—July/*P. sylvestris,* *Oct_TC*—October/*T. cordata*, *Jul_BP*—July/*B. pendula*, *Jul_TC*—July/*T. cordata*, *Oct_BP*—October/*B. pendula*. For abbreviations of mite taxa see Table 6 in Appendix 3

**Fig. 5:**
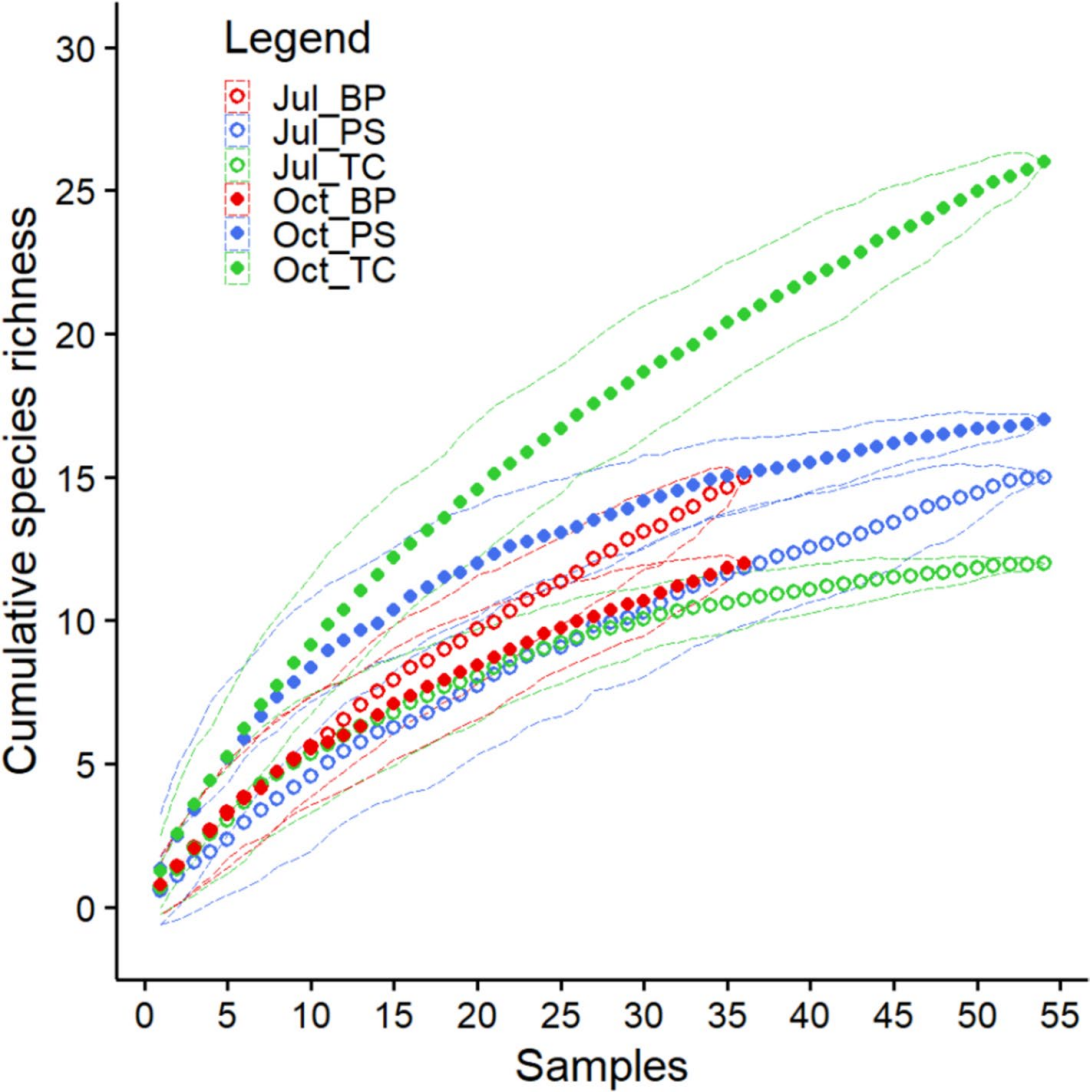
Cumulative species richness (data presented as mean values) for each month/habitat examined. *Oct_PS*—October/*P. sylvestris*, *Jul_PS*—July/*P. sylvestris*, *Oct_TC*—October/*T. cordata*, *Jul_BP*—July/*B. pendula*, *Jul_TC*—July/*T. cordata*, *Oct_BP*—October/*B. pendula*

**Fig. 6:**
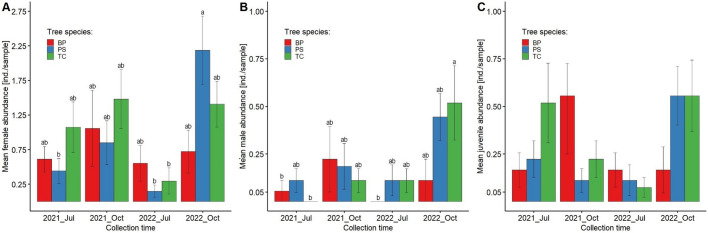
Mean female (**A**), male (**B**) and juvenile (**C**) abundance of mesostigmatid mite communities across four sampling periods (July, October; 2020, 2021) in pure stands on post-agricultural lands (Opole Forest District).. *Oct*—October, *Jul*—July, *BP*—*B. pendula*, *PS*—*P. sylvestris*, *TC*—*T. cordata*

Most mite individuals belonged to the Parasitidae (111 ind.), Laelapidae (95) and Veigaiidae (70) families (Table 1). The most abundant species were *Hypoaspis* (*Gaeolaelaps*) *aculeifer* (21.6% of all recorded mites), *Veigaia nemorensis* (7.8%) and *Trachytes aegrota* (7.0%). Each of these species was most abundant in the *T. cordata* stand in October. Only two species occurred in each studied variant – *Veigaia nemorensis* and *Hypoaspis aculeifer*, while 15 mite species were unique for one variant (Fig. 4, Table 6 in Appendix 3). The cumulative species richness for *T. cordata* stand in October and July exceeded those for other examined month/habitat combinations (Fig. 5). The lowest juvenile abundance was recorded in *T. cordata* stand in July 2022 (0.07 ± 0.05 ind.; 2.4% of all juveniles). However, the number of juveniles was not statistically different among studied habitats and collection months (Fig. 3C). Interestingly, most females (2.19 ± 0.49 ind.; 22.2% of all females) were reported in *P. sylvestris* stand in October 2022, while the lowest female abundance was found in *P. sylvestris* stand in July of the same year (0.15 ± 0.09) (Fig. 6A). Males were the most numerous in the *T. cordata* stand in October 2022 (0.52 ± 0.20 ind.; 28.0% of all males). Moreover, no males were recorded in July 2022 in *T. cordata* and *B. pendula* stand (Fig. 4B). Furthermore, the most juveniles were found in *P. sylvestris* (0.56 ± 0.19 ind.; 18.1% of all juveniles) and *T. cordata* stand (0.56 ± 0.15; 18.1%) in October 2022, as well as in *B. pendula* stand in October 2021 (0.56 ± 0.30; 12.1%) (Fig. 6C).

The largest number of *H. aculeifer* females was found in samples taken in October from the *P. sylvestris* and *T. cordata* stands (in both cases there were 22 individuals from two years, which accounted for 81.48% of all individuals of this species in the variant). In contrast, the largest number of *H. aculeifer* juveniles was found in samples taken in July from the *T. cordata* stand (6 ind.; 42.86%). Similarly, in the case of *V.nemoresnis*, the most females were found in samples collected in October from the *T.cordata* stand (10 ind.; 83.33%), while the most juvenile stages were reported in July in the *T. cordata* stand (4 ind.; 66,67%). No *T. aegrota* individuals were found in *B. pendula* stands, while the highest number of individuals of this species was reported in the *T. cordata* stands in October (35.71% of all *T. aegrota* individuals in total).

## Discussion

### Seasonal changes in mite communities

Generally, we found a significantly higher abundance of Mesostigmata mites in October than in July, as we expected from the results of other studies (Fujii and Takeda 2017). It alludes to the study by Kaczmarek et al. (2011), highlighting that soil moisture is the main factor that allows soil mite assemblage to develop. Indeed, in the climatic conditions of Poland, lower abundance in July may be explained by higher temperature and rapidly decreasing soil moisture in summer, which causes a decrease in mite density. With the end of summer, the temperature remains high, but soil moisture increases. Consequently, this causes an increase in soil mite density (Salmane 2000). The same relationship also applied to species richness and diversity of soil acarofauna. Similar importance of precipitation and temperature for the soil mite community structure was also revealed by Kamczyc et al. (2022), who reported that the abundance of mesostigmatid mites in decomposing litter of broadleaved and coniferous temperate tree species was positively correlated with the temperature of the sampling month and negatively correlated with the temperature of the month before sampling. The difference with our study was that the abundance shown in summer (July) was higher than in autumn (October). Species richness and diversity showed only slight changes between consecutive months of the growing season (April–October).

An interesting result of our study is the increase in the proportion of juvenile instars in summer. They are considered to be more sensitive than adult mite instars to temperature increases and humidity decreases, which is associated *inter alia* with a lower degree of body sclerotization (Huhta and Hänninen 2001). Adult mites should be more resistant than juveniles to water loss and less susceptible to changes in temperature and humidity (Urbanowski et al. 2021). An explanation for our result may be the overlap of the period of high mortality with the hatching of mesostigmatid juveniles. As demonstrated by Kaczmarek et al. (2011), under similar climatic conditions, there are two density peaks for mesostigmatid mites—in January and at the turn of spring and summer.

Most identified individuals belonged to the Parasitidae, Laelapidae and Veigaiidae families. Mites of the Parasitidae family are found in all types of forests, meadows, bogs, and farmland. They mainly inhabit the litter but also the humus layer. Their main prey are springtails and other mites, such as Oribatida and Astigmata (Błoszyk 2008). The Laelapidae family includes, among others, large predatory species of the *Hypoaspis* genus. *Hypoaspis* (*Geolaelaps*) *aculeifer* is a common pioneer species (Wissuwa et al. 2012), also found in large numbers on former farmland. *Hypoaspis aculeifer* is a species that *inter alia* preys on springtails (Cortet et al. 2003). Veigaiidae family contains species specialised in feeding on springtails, but may also feed on other soil fauna (Koehler 1999). They primarily inhabit forest litter in the Palearctic but are also found in meadows and agricultural land (Błoszyk 2008). Together with the mites of the Parasitidae family, they form the dominant group of predators in the Mesostigmata order (Koehler 1997; Błoszyk 2008; Skorupski 2008; Kazemi et al. 2013). *Veigaia nemorensis* is a common species in the litter and upper soil layers on former farmland and even on degraded or post-industrial sites (Skorupski et al. 2013; Manu et al. 2017). *Veigaia nemorensis*, in contrast to the general characteristics of the Veiigaidae family, mainly preys on bacterial and root-feeding nematode species (Manu et al. 2017).

### Tree species impact

The differences in litter impact of tree species such as *P. sylvestris*, *B. pendula and T. cordata* on mesostigmatid mite assemblages were compared by Kamczyc et al. (2019) and Urbanowski et al. (2021). Kamczyc et al. (2019) showed that tree litter only affected abundance, while there was no influence on species richness and diversity of mesostigmatid mite assemblages. In addition, they indicated a positive effect of *P. sylvestris* litter comparing to *T. cordata* litter on soil mite density. In contrast, Urbanowski et al. (2021) showed no significant differences in the effect of *P. sylvestris* and *B. pendula* litter on the abundance, species richness and diversity of mesostigmatid mite communities. Our study did not show differences in the influence of individual tree species on the soil Mesostigmata. However, when comparing mite assemblages in summer and autumn, we observed high differences in mite abundance, species richness and diversity between the studied stands. Our results indicate that the most extreme abundance values ​​were found in *P. sylvestris* stand, which provided the most favourable conditions for the soil fauna in autumn and the least favourable in summer. This effect may be explained by the highest water absorption capacity of *P. sylvestris* litter, a coniferous species, with the greatest decreases in moisture during dry months (Zhou et al. 2018). Interestingly, Ilek et al. (2024) revealed that forest litter with a low C:N ratio and a predominant share of oak leaves achieved the greatest water storage capacity, while litter with a high C:N ratio and a predominant share of *P. sylvestris* needles had the lowest water storage capacity. This is consistent with our results because the C:N ratio was the lowest in the *P. sylvestris* stand and the highest in the *T. cordata* stand. Also in the *B. pendula* stand, mite abundance and diversity were significantly lower in summer than in *P. sylvestris* stand in autumn. We assume that this results from the fact that *B. pendula* stands are less able to protect the soil in summer from heating and drying out (Jonczak et al. 2020).

### Interaction between soil parameters and mite communities

Our study revealed that Fe content in soil affected abundance, species richness and diversity of mite communities—the biodiversity decreased as soil Fe concentration increased. The negative effects of this nutrient on soil fauna were also noted by Huot et al. (2018) and Rusek and Marshall (2000). An increase in soil Fe concentration may limit the development of springtails, reduce manganese uptake, and stress soil organisms through the energy-demanding detoxification process (Rusek and Marshall 2000). Christophe et al. (2014) proved that the concentration of elements such as Fe in the rhizosphere solution and the soil environment changes with the seasons and is highest in autumn. Widowati and Sukristyonubowo (2019) showed that Fe becomes less toxic to plants when the soil pH is lower. In turn, Thoms and Gleixner (2013) observed that soil pH is lower in autumn than in summer.

Also, the collection month significantly affected the abundance, species richness and diversity of mesostigmatid mite communities. Changes in weather conditions with the ongoing seasons appear to be crucial to the density and diversity of soil fauna communities. This is not surprising because mites are ectothermic organisms, depending on climatic conditions such as temperature and precipitation (Thakur et al. 2018). This result was also confirmed by other studies conducted in central Europe (Urbanowski et al. 2021; Kamczyc et al. 2022). Additionally, species richness and diversity of mesostigmatid mites increased with litter nitrogen content, which may be associated with a higher biomass and species richness of microorganisms and nematodes (Kaneko and Salamanca 1999; Sánchez-Moreno et al. 2009; Renčo et al. 2020). Interestingly, in our study, the abundance of mites was not influenced by litter nitrogen content, which is correlated with the type of litter (Cornwell et al. 2008; Horodecki and Jagodziński 2019). It corresponds to the conclusions of studies conducted by Seniczak et al. (2018) on moss mite (Oribatida) and mesostigmatid mite communities. They reported that nutrient-rich habitats generate high species diversity but low densities of soil mites. This may mean that poorly fertile habitats have the opposite trend in mite community structure.

## Conclusions

In conclusion, we revealed that different tree species growing on post-agricultural lands shape various soil conditions, which change between the summer and autumn seasons. Large fluctuations in soil mite abundance, species richness and diversity between *P. sylvestris* and *B. pendula* stands in summer and autumn may depend on tree species and season, which may constitute proof for using mixed stands during afforestation of post-agricultural lands. Seasonal change in the proportion between instars was unclear, but contrary to expected results, the proportion of young individuals increased in summer.

## Data Availability

We declare all data is being provided within this manuscript.

## Appendix 1

See Table 4.

**Table 4:** Stand characteristic for each study plot

Study plot	Forest stand	Age of forest stand [Years]	Number of trees [N/ha]	Avg. DBH [mm]	SD Dbh [mm]	Avg. height [m]	SD height [m]	Basal area [m^2^/ha]	Volume of gross timber [m^3^/ha]	Coverage and species of tree layer [%]	Coverage and species of shrub layer [%]	Coverage and species of vascular ground layer [%]	Coverage and species of bryophytes ground layer [%]
1	*T. cordata*	22	3500	**96**	28	**10.0**	1.0	**27.5**	**95**	100 T*. cordata*	10 T*. cordata*	30 V*. hederifolia*	–
2	*T. cordata*		2900	**104**	29	**11.1**	1.1	**26.4**	**115**	100 T*. cordata*	10 T*. cordata*	50 V*. hederifolia*	–
3	*T. cordata*		3950	**80**	23	**8.3**	1.5	**21.6**	**52**	100 T*. cordata*	20 T*. cordata*	40 V*. hederifolia*	–
4	*B. pendula*		2350	**121**	29	**16.5**	2.0	**28.5**	**224**	80 *B. pendula*	–	40 *S.canadensis*, 20 *G. aparine*, 10 *U. dioica*, 10 *C. arundinacea*, 10 V*. hederifolia*	30* P. schreberi*
5	*B. pendula*		1700	**123**	32	**15.1**	2.0	**21.4**	**159**	70 *B. pendula*	10 *C. monogyna*	40 *S. canadensis*, 20 V*. hederifolia*, 10 *C. arundinacea*, 10 *U. dioica*, 10 *G. aparine*	10 *P. schreberi*
6	*P. sylvestris*		2500	**144**	38	**14.7**	0.6	**43.5**	**307**	90 *P. sylvestris*	–	–	50 *P. schreberi*
7	*P. sylvestris*		2850	**124**	38	**13.7**	1.0	**37.7**	**253**	90 *P. sylvestris*	–	–	40 *P. schreberi*
8	*P. sylvestris*		2800	**127**	37	**13.8**	1.2	**38.2**	**260**	100 *P. sylvestris*	–	–	10 *P. schreberi*

*SD*—standard deviation, *T. cordata—Tilia cordata, B. pendula—Betula pendula, P. sylvestris—Pinus sylvestris, C. monogyna—Crataegus monogyna, V. hederifolia—Veronica hederifolia, S. canadensis—Solidago canadensis, G. aparine—Galium aparine, U. dioica—Urtica dioica, C. arundinacea—Calamagrostis arundinacea, P. schreberi—Pleurozium schreberi*

## Appendix 2

See Table 5.

**Table 5:** Soil properties for each study plot

Plot	Tree species	Soil texture	Litter pH	Litter thickness (cm)	Soil pH	CaCO_3_ (%)	SOM (%)	Corg (%)	Bulk density (g/cm^3^)	Soil moisture (%)	Soil N (%)	Litter N (%)	Soil Mg (%)	Litter Mg (%)	Soil Ca (%)	Litter Ca (%)
1	TC	S	5.36	1.5	5.03	0	3.41	1.98	1.631	8.0	0.190	1.819	0.1919	0.2075	0.0220	0.1874
2	TC	S	5.68	1.5	4.66	0	2.43	1.41	1.308	7.1	0.238	1.907	0.1930	0.2095	0.0029	0.1952
3	TC	S	5.47	1.4	4.72	0	2.09	1.21	1.436	8.1	0.221	1.503	0.1883	0.2104	0.0100	0.1936
4	BP	S	5.68	1.7	4.11	0	1.89	1.10	1.404	4.6	0.170	1.486	0.1824	0.2078	0.0011	0.1812
5	BP	S	5.63	1.6	4.24	0	2.03	1.18	1.411	4.5	0.150	1.605	0.1858	0.0000	0.0003	0.0000
6	PS	S	4.63	2.9	3.51	0	1.79	1.04	1.498	1.9	0.143	1.513	0.1841	0.2021	0.0014	0.1787
7	PS	S	4.51	3.3	3.52	0	1.14	0.66	1.436	1.3	0.129	1.601	0.1890	0.2020	0.0036	0.1826
8	PS	S	4.9	2.9	4.02	0	1.39	0.81	1.547	3.8	0.146	1.411	0.1833	0.2025	0.0031	0.1669

Plot	Soil Na (%)	Litter Na (%)	Soil K (%)	Litter K (%)	Soil Fe (mg/kg)	Litter Fe (mg/kg)	Soil Cd (mg/kg)	Litter Cd (mg/kg)	Soil Pb (mg/kg)	Litter Pb (mg/kg)	Soil Mn (mg/kg)	Litter Mn (mg/kg)	Soil Zn (mg/kg)	Litter Zn (mg/kg)	Soil Cu (mg/kg)	Litter Cu (mg/kg)
1	0.0024	0.0042	0.0137	0.0792	333.05	141.36	0.25	0.15	12,41	5.79	276.03	404.37	13.59	32.66	13.59	32.66
2	0.0027	0.0045	0.0068	0.0660	339.56	237.62	0.22	0.54	15,62	7.57	149.23	406.31	14.38	66.82	14.38	66.82
3	0.0029	0.0089	0.0146	0.0740	334.26	237.96	0.16	0.42	11,91	2.29	200.75	415.81	10.23	46.08	10.23	46.08
4	0.0023	0.0040	0.0060	0.0470	344.38	252.53	0.09	0.68	13,09	9.11	269.04	434.02	4.79	78.26	4.79	78.26
5	0.0029	0.0000	0.0067	0.0000	350.59	0.00	0.12	0.00	14,29	0.00	273.45	0.00	4.21	0,00	4.21	0.00
6	0.0025	0.0034	0.0044	0.0603	331.93	163,26	0.12	0.60	10,15	5.48	155.42	427.06	5.74	40.01	5.74	40.01
7	0.0023	0.0036	0.0148	0.0501	348.07	181.90	0.12	0.53	8,82	5.09	144.00	424.27	6.83	31.61	6.83	31.61
8	0.0019	0.0040	0.0049	0.0601	346.33	179.07	0.12	0.39	8,24	3.33	106.77	395.76	5.93	22.00	5.93	22.00

*TC—Tilia cordata*, *BP—Betula pendula*, *PS—Pinus sylvestris,* *S—Sand,* *SOM*—soil organic matter, *Corg*—organic carbon

## Appendix 3

See Table 6.

**Table 6:** Network statistics for mesostigmatid mite taxa, describing their affiliation to habitats and specialisation

No	Mite taxa	Abbreviation	Number of habitats	Proportion of habitats	Species specificity index d′
1	*Alliphis halleri* (Canestrini & Canestrini, 1881)	Al_ha	1	0.17	1.000
2	*Amblyseius* spp.	Am_sp	2	0.33	0.817
3	*Antennoseius bacatus* Athias-Henriot, 1961	An_ba	2	0.33	0.633
4	*Arctoseius venustulus* (Berlese, 1917)	Ar_ve	2	0.33	0.633
5	*Asca aphidioides* (Linnaeus, 1758)	As_ap	1	0.17	1.000
6	*Eugamasus cavernicolus* Trägårdh, 1912	Eu_ca	1	0.17	1.000
7	*Gamasellodes bicolor* (Berlese, 1918)	Ga_bi	1	0.17	1.000
8	*Gamasellus montanus* (Willmann, 1936)	Ga_mo	2	0.33	0.633
9	*Hypoaspis* (*Geolaelaps*) *aculeifer* (Canestrini, 1883)	Hy_ac	6	1.00	0.305
10	*Hypoaspis* spp.	Hy_sp	2	0.33	0.714
11	*Hypoaspis* (*Geolaelaps*) *praesternalis* (Willmann, 1949)	Hy_pr	1	0.17	1.000
12	*Leptogamasus succineus* Witaliński, 1973	Le_su	2	0.33	0.785
13	*Leptogamasus tectegynellus* (Athias Henriot, 1967)	Le_te	1	0.17	1.000
14	*Macrocheles montanus* (Willmann, 1951)	Ma_mo	4	0.67	0.466
15	*Macrocheles* spp.	Ma_sp	1	0.17	1.000
16	*Olodiscus minima* (Kramer, 1882)	Ol_mi	4	0.67	0.418
17	*Oodinychus ovalis* (C.L.Koch, 1839)	Oo_ov	3	0.50	0.592
18	*Pachyseius humeralis* (Berlese, 1910)	Pa_hu	1	0.17	1.000
19	*Paragamasus conus* (Karg 1971)	Pa_co	5	0.83	0.449
20	*Paragamasus jugincola* (Athias Henriot, 1967)	Pa_ju	1	0.17	1.000
21	*Paragamasus runcatellus* (Berlese, 1903 sensu Karg 1971)	Pa_ru	4	0.67	0.361
22	*Paragamasus* spp.	Pa_sp	4	0.67	0.471
23	*Paragamasus vagabundus* (Karg, 1968)	Pa_va	5	0.83	0.783
24	*Pergamasus barbarus* (Berlese, 1904)	Pe_ba	2	0.33	0.785
25	*Pergamasus crassipes* (Linnaeus, 1758)	Pe_cr	2	0.33	0.633
26	*Pergamasus mediocris* Berlese, 1904	Pe_me	1	0.17	1.000
27	*Pergamasus septentrionalis* (Oudemans, 1902)	Pe_se	4	0.67	0.375
28	*Pergamasus* spp.	Pe_sp	3	0.50	0.500
29	*Rhodacarus mandibularis* (Berlese, 1921)	Rh_ma	1	0.17	1.000
30	*Trachytes aegrota* (C.L.Koch, 1841)	Tr_ae	4	0.67	0.374
31	*Veigaia cerva* (Kramer, 1876)	Ve_ce	1	0.17	1.000
32	*Veigaia decurtata* (Athias Henriot, 1961)	Ve_de	3	0.50	0.669
33	*Veigaia exigua* (Berlese, 1916)	Ve_ex	5	0.83	0.432
34	*Veigaia nemorensis* (C.L.Koch, 1839)	Ve_ne	6	1.00	0.302
35	*Veigaia planicola* (Berlese, 1892)	Ve_pl	2	0.33	0.633
36	*Vulgarogamasus kraepelini* (Berlese, 1904)	Vu_kr	1	0.17	1.000
37	*Zercon peltatus peltatus* (C.L.Koch, 1836)	Ze_pe	4	0.67	0.416
38	*Zercon hungaricus* (Sellnick, 1958)	Ze_hu	1	0.17	1.000
39	*Zercon triangularis* (C.L.Koch, 1836)	Ze_tr	1	0.17	1.000
